# The complexities of joy: a qualitative study of joy cultivation, loss of joy, and happiness in British adults

**DOI:** 10.1080/17482631.2025.2508946

**Published:** 2025-05-24

**Authors:** Maria Roberts, Richard Appiah

**Affiliations:** a Department of Psychology, Faculty of Health and Life Sciences, Northumbria University, Newcastle Upon Tyne, UK; b College of Health Sciences, University of Ghana, Accra, Ghana; c Department of Psychology, University of Johannesburg, Johannesburg, South Africa

**Keywords:** Joy, emotional well-being, mental health, happiness, positive psychology, United Kingdom

## Abstract

**Introduction:**

Joy is a fundamental yet underexplored emotion that plays a critical role in well-being and happiness. Despite its significance, little research has examined how individuals cultivate, experience, and lose joy, particularly in relation to its interplay with other emotions and broader aspects of well-being. This study explored the subjective and contextual dimensions of joy among UK adults, including how it is cultivated, lost, and related to the broader aspects of well-being.

**Methods:**

Using an exploratory qualitative design, semi-structured interviews were conducted with 14 adults aged 28–59 years. Thematic analysis was employed to identify patterns in participants’ experiences of joy, its influences, and impact on well-being.

**Results:**

Four key themes were constructed: The Dynamics of Joy in Everyday Life, Determinants of Joy: Influences and Barriers, Impact of Joylessness: Coping Behaviours, and Strategies for Cultivating Joy. Joy was perceived as a distinct, profound emotion, often intertwined with gratitude and love. Barriers to joy included negative relationships, societal pressures, and emotional burdens, whereas fostering positive relationships, engaging with nature, and cultivating self-awareness were identified as key strategies for sustaining joy.

**Discussion:**

Findings highlight joy as a complex emotion, distinct from happiness and shaped by individual, relational, and cultural contexts. The study offers insight into joy’s emotional and psychological impact, the challenges of its loss, and the enduring value of joyful memories. Participants also shared practical suggestions for cultivating joy, which may inform well-being initiatives in community, educational, or therapeutic settings.

## Introduction

Joy is a complex and essential positive emotion that plays a pivotal role in human wellbeing, often experienced as a transient state but also reflecting enduring individual differences in emotional disposition (Watkins et al., 2018). While often used interchangeably with happiness, joy is conceptually distinct. Joy is typically described as a more intense, transcendent, and deeply felt emotion, often arising spontaneously in response to meaningful experiences or connections. In contrast, happiness is generally viewed as a broader, more stable evaluative state associated with life satisfaction (Damasio, 2003; Johnson, 2020a; Watkins et al., 2018). Unlike happiness, which can be cultivated through goal achievement or positive life circumstances, joy may emerge in unexpected moments, even amid hardship. This distinction underscores the need to explore joy in its own right, particularly given its foundational role in emotional flourishing and psychological resilience (Emmons, 2020; Fredrickson, 2001; King, 2020). Despite its significance, joy remains underexplored in empirical research. The Broaden-and-Build Theory posits that even brief experiences of joy can foster the long-term development of social, cognitive, and physical resources, counteracting the lingering effects of negative emotions (Fredrickson, 2001, 2013; Waugh, 2020). Joy is also central to social bonding and connection (Johnson, 2020b), with some scholars describing it as a powerful antidote to hatred and division (Mathewes, 2020).

Although often perceived as a hedonic experience, joy is deeply intertwined with the sense that life is meaningful and fulfilling, an idea closely linked to eudaimonic wellbeing, which refers to well-being derived from living in alignment with one’s values, purpose, and personal growth (Johnson, 2020b; Mathewes, 2020; Ryan & Deci, 2021). Neurologically, joy has been associated with integrated brain function (Davidson, 2012; Vaillant, 2008) and healthy mental states (Siegel & Drulis, 2023). Joy has traditionally been the subject of philosophical and theological reflection and debate, particularly concerning its moral, spiritual, and existential significance (Krumrei-Mancuso, 2020). These traditions have explored whether joy is a fleeting pleasure, a cultivated virtue, or a spiritual state of grace, often with differing conclusions. Such debates contribute to the conceptual complexity of joy. In psychological research, this complexity is further evident in findings that joy can sometimes evoke vulnerability, shame, or coexist with sorrow, rather than merely reflecting unambiguous positivity (Johnson, 2020b; Vaillant, 2008). In this study, we distinguish between emotional experiences of joy (affective responses such as warmth, excitement, or awe) and mental experiences of joy, which refer to internal cognitive states such as clarity, perspective, insight, or mental stillness (Davidson & Schuyler, 2015; Siegel & Drulis, 2023). Research suggests that cultivating joy may be more effective for promoting resilience and long-lasting wellbeing than the pursuit of happiness alone (King, 2020; Mathewes, 2020).

Despite the growing interest in positive emotions within the field of positive psychology (Emmons, 2020; King, 2020), joy remains relatively underexplored compared to happiness. Empirical studies have only recently begun to investigate joy in greater depth (Watkins et al., 2018), leaving significant gaps in our understanding of its subjective, contextual, and personal dimensions. Many individuals report experiencing joy infrequently, and the factors that influence its manifestation vary widely (Johnson, 2020a; Underwood, 2020). Researchers argue that understanding the appraisal structure and expressions of joy is crucial to advancing our knowledge of emotional wellbeing (Gilbert et al., 2014).

While quantitative research has provided valuable insights into the effects and correlates of joy (Watkins et al., 2018), it has struggled to induce joy reliably in experimental settings (Catalino & Tov, 2022). This challenge underscores the need for qualitative inquiry, which offers a more nuanced exploration of the lived experiences and subjective narratives of joy. Qualitative research provides the opportunity to examine how joy emerges, how it is sustained, and how it is lost in the context of modern life.

If joy is indeed central to building resilience and fostering wellbeing (Emmons, 2020; Fredrickson, 2001), then developing interventions that enhance joy within individuals and communities could offer meaningful ways to navigate adversity and uncertainty (Johnson, 2020a; Krumrei-Mancuso, 2020; Underwood, 2020). Understanding the personal and environmental conditions that cultivate or inhibit joy is a critical first step (Emmons, 2020; Ryan & Deci, 2021; Watkins et al., 2018). Theoretical perspectives such as King’s (2020) view of joy as a virtue, defined as a cultivated capacity to recognize and respond to the good in life, even amid adversity, and Fredrickson’s (2001) Broaden-and-Build Theory highlight the broader implications of joy for mental health, resilience, and long-term flourishing. Joy is also intricately connected to life narratives (King, 2020), suggesting that qualitative approaches can reveal the deeper, subjective dimensions of this emotion. A positive psychology framework that emphasizes strengths, resilience, and wellbeing (Rashid & Seligman, 2018; Seligman & Csikszentmihalyi, 2000) is well suited to explore these narrative dimensions of joy, providing insights into how joy can be nurtured and sustained in everyday life.

This study aimed to explore the lived experience of joy among adults in the United Kingdom. Specifically, the objectives were to: (1) examine how individuals experience, express, and interpret joy in everyday life; (2) identify the personal, relational, and contextual factors that influence the cultivation or loss of joy; (3) explore how joy relates to happiness and broader well-being; and (4) investigate strategies that individuals and communities use to foster and sustain joy. Understanding these processes will be crucial for enhancing resilience and preventing the diminishment of joy in a world increasingly characterized by stress and uncertainty (Emmons, 2020; Underwood, 2020; Watkins, 2020).

This research will contribute to the growing body of literature on emotional wellbeing, offering practical insights for future interventions aimed at promoting joy, happiness, and resilience in diverse populations. This study sets out to deepen our understanding of joy to inform future research, practice, and policy, ultimately contributing to a more joyful and flourishing society.

## Methods

### Study design

This study adopted an exploratory, phenomenological, and descriptive qualitative research design (Creswell, 2014), chosen to capture participants’ lived experiences and subjective conceptualizations of joy, including its cultivation, loss, and associated strategies. Such a design is particularly suited to exploring the nuances of emotional experiences that are complex, context-dependent, and resistant to quantitative measurement (Braun & Clarke, 2022). The critical realist approach underlying the study (Braun & Clarke, 2006) allowed for the exploration of participants’ constructed meanings within their broader social realities, aligning with the study’s objectives to understand how joy is cultivated and lost in everyday life.

### Data collection instrument and procedure

Data were collected using semi-structured interviews, a method selected for its flexibility and capacity to elicit in-depth responses while ensuring that key topics were consistently explored across participants (Kallio et al., 2016). An interview schedule, designed in line with the research objectives and informed by existing literature, guided the interviews (see Supplementary Table S1). The schedule included open-ended questions that explored participants’ definitions of joy, its expression, its connection to happiness, and strategies for its cultivation.

Interviews were conducted online via Microsoft Teams, which was selected for its accessibility, security, and ability to record high-quality video and audio data remotely. The use of virtual platforms for qualitative research has been validated as a practical and effective method for in-depth data collection, particularly in cases where geographical dispersion of participants could limit face-to-face interactions (Gray et al., 2020). Apple Voice Recorder served as a backup audio recording tool to ensure data security in case of technical failures. An initial broad question, “*Can you describe a specific moment that you experienced joy and happiness in your life? How did you process and express that joy?*” were posed. Probes were employed throughout the interviews to deepen participants’ reflections, clarify meanings, and encourage elaboration. Each primary question in the interview schedule was accompanied by at least one structured, tailored probe designed to elicit richer, context-specific narratives and uncover the relational, cultural, and personal dynamics of joy (see Supplementary Table S1). Examples include prompts such as, “*In your opinion, how might cultural or societal influences shape an individual’s understanding and expression of joy?*” and “*How do social connections and relationships impact your ability to cultivate and sustain joy?*” In addition to these guide-based probes, spontaneous follow-up questions were used flexibly during the interviews to pursue unexpected or emergent themes as they arose (Seidman, 2013).

Prior to the interviews, participants were sent the interview questions to encourage thoughtful reflection and help them feel more comfortable and prepared. The sample consisted of 14 participants (2 male, 12 female), aged 28 to 59 (*M* = 49, SD = 8), predominantly White British (*n* = 13), and mainly residing in northern England (*n* = 12), with one participant each from London and Wales. Based on occupation, the sample reflected a moderate to high socio-economic status. Educational backgrounds were diverse, ranging from GCSE/O Level (Level 2) to doctoral-level qualifications (Level 8) (see Table I). Participants were recruited through convenience sampling via social media platforms (Facebook, X, and WhatsApp), a common method in exploratory qualitative research to reach a broad demographic (Etikan et al., 2016). Each interview lasted an average of 49 minutes, with durations ranging from 30 to 73 minutes. Data collection continued until theoretical saturation was reached with the 14th interview, consistent with Baker and Edwards (2012) recommendation that a sample size of 12–60 participants is sufficient for qualitative exploration. Theoretical saturation was determined through an iterative review of developing codes and constructed themes after each interview. Specifically, saturation was considered to have been reached when no new codes, concepts, or analytic insights were identified across three consecutive interviews, and when the data collected were deemed sufficient to fully address the study’s aims (Fusch & Ness, 2015). The decision was also guided by the richness, depth, and redundancy of participant narratives across the key domains explored, such as definitions of joy, its loss, and strategies for cultivation. At this point, additional interviews were no longer yielding novel insights or altering the thematic framework, indicating that saturation had been achieved. Probing questions were used during the interviews to explore preliminary patterns and deepen the developing thematic insights, adding depth and ensuring data richness (Gill et al., 2008).

**Table I.** Participant characteristics (n = 14).

Demographic Category	Subcategory	n
Gender	Male	2
	Female	12
Age Range	28–59	—
Mean Age (SD)	49 (8)	—
Education Level	Level 2 – GCSE/O Level	2
	Level 3 – A Levels	2
	Level 4 – Certificate of Higher Ed.	1
	Level 6 – Bachelor’s Degree	1
	Level 7 – Master’s Degree	6
	Level 8 – Doctorate or Equivalent	2
Ethnicity	White British	13
	White Other	1
Employment	Carer/Homemaker	2
	Working in Education	4
	Retired	2
	Manager/Programme Lead	3
	Design Engineer	1
	Self-Employed	1
	Letting Agent	1
Marital Status	Married	11
	Single	3
Religion/Spirituality	Spiritual	6
	Religious	1
	Unsure	2
	Not Religious or Spiritual	5

### Researcher reflexivity and positionality

Reflexive thematic analysis positions the researcher as an active and interpretive agent in the analytic process, shaping, not discovering, meaning through their interactions with the data (Braun & Clarke, 2022). In this study, the first author drew upon her professional background in education, psychotherapy, and community work with children and families. She identifies as a British, White, middle-aged, heterosexual woman with both working- and middle-class roots. Her humanistic values, prior use of strengths-based approaches, and personal engagement with emotional well-being sensitized her to the study’s focus on joy.

Joy held emotional and professional significance for the first author. While she approached the study with a belief in its relevance to resilience and flourishing, she remained reflexively attuned to the ways in which her assumptions, values, and cultural lens shaped the questions asked, the data attended to, and the themes constructed. Reflexivity was supported through ongoing journaling, analytic memos, and critical dialogue with the co-author. These practices helped surface implicit biases and contributed to analytic depth. Rather than treating positionality as a bias to be controlled, the researcher approached it as a resource that enriched meaning-making (Goundar, 2025). The themes presented in this paper were thus developed through iterative, situated engagement between participant narratives and the researcher’s interpretive lens (Braun et al., 2023).

### Data analysis

Data were analysed using reflexive thematic analysis (TA), as outlined by Braun and Clarke (2006, 2022), which is widely recognized for its flexibility and robustness in identifying and analysing patterns within qualitative data. Reflexive TA emphasizes the active role of the researcher in the analysis process, with the researcher bringing their reflexive insights to the interpretation of the data (Clarke, 2021). This method was particularly suitable for this study, as it allowed for an iterative and inductive approach to theme development, ensuring the analysis remained grounded in participants’ lived experiences.

The analysis began with familiarization, during which the researcher listened to the audio recordings and manually checked and anonymized the transcriptions. Initial coding was conducted using NVivo software (v12) to support systematic organization, data segmentation, and open coding. NVivo was used to generate initial descriptive codes and to explore frequency and co-occurrence of concepts across transcripts, providing a structured and transparent approach to handling the qualitative dataset (Bazeley & Jackson, 2013). As the analysis progressed into a more interpretive stage, the researcher transitioned to manual coding. This allowed for greater immersion in the data and facilitated the development of broader analytical themes by visually clustering and comparing codes in ways that extended beyond NVivo’s automated visualization features. The manual mapping process involved physically arranging codes and subthemes, enabling a more reflexive, iterative refinement of the developing thematic structure. Themes were iteratively refined through repeated engagement with the full interview transcripts to ensure that each theme was grounded in participants’ narratives, accurately reflected the data, and was analytically distinct from other themes. This process involved checking that themes were supported by sufficient and varied quotes, revisiting the context of specific responses, and ensuring thematic coherence across the dataset. A final thematic map was produced, presenting a comprehensive narrative of participants’ experiences of joy, its cultivation, and its loss.

We employed reflexive thematic analysis and engaged in regular reflexive journaling throughout the process to critically examine their positionality, assumptions, and interpretations (Braun et al., 2023). In addition, peer debriefing was undertaken with the second author reviewing selected transcripts, coding patterns, and theme development to ensure consistency and credibility of the analytic process. This collaborative peer review acted as a form of validation by challenging interpretations and enhancing analytical transparency. Verbatim quotes from the interviews were included in the final report to support the findings and preserve the authenticity of participants’ voices. These quotes were selected to illustrate key themes and provide insight into the subjective and contextual dimensions of joy as expressed by participants.

### Ethical considerations

Ethical approval for this study was granted by the Northumbria University Ethics Committee (2023–6267–5582). Participants were informed of their right to withdraw from the study at any stage without any negative consequences, which was reiterated before the interviews commenced. All participants provided written informed consent, ensuring they fully understood the nature of the research, their participation, and how their data would be used (Wiles, 2012).

Confidentiality was maintained throughout the study. Interviews were conducted in private virtual settings to protect participants’ privacy, and all personally identifying information was anonymized during transcription. Participants’ names were replaced with pseudonyms, and all potentially identifying details were redacted to protect their anonymity (Saunders et al., 2015). Data were securely stored on password-protected devices, adhering to GDPR guidelines (Clark & Albris, 2020).

## Results

The analysis identified four key themes, each with corresponding subthemes that illuminate the multifaceted nature of joy in the lives of UK adults. The first theme, *The Dynamics of Joy in Everyday Life*, explores how joy is experienced as a paradox, a physical and emotional sensation, and a communal phenomenon. The second theme, *Determinants of Joy: Influences and Barriers*, examines how joy is shaped by negativity, positivity, cultural and familial influences, and the role of connection. The third theme, *Impact of Joylessness: Coping Behaviours*, focuses on how joy impacts mental health and happiness, particularly through coping with joylessness and the enduring influence of joyful memories. Finally, the fourth theme, *Strategies for Cultivating Joy*, highlights the role of education, conversations, and revitalizing community connections in fostering joy.

Table II presents the superordinate and subordinate themes.

**Table II.** Superordinate and subordinate themes.

Superordinate theme	Sub-theme
The Dynamics of Joy in Everyday Life	Joy as a Paradox The Physical and Emotional Symphony of Joy The Communal Nature of Joy
Determinants of Joy: Influences and Barriers	The Influence of Positivity and Negativity Cultural and Familial Influences The Role of Connection Navigating Life’s Burdens
Impact of Joylessness: Coping Behaviours	Coping Behaviours in Response to Joylessness Joy’s Enduring Influence: Mental Health and Memory
Strategies for Cultivating Joy	Joy Education Conversations About Joy Revitalising Community Connections

## The dynamics of joy in everyday life

This main theme addresses the first objective of the study, exploring how adults in the UK experience, interpret, express, and lose joy. It encapsulates the complex, multifaceted nature of joy as articulated by participants. The three sub-themes describe the paradoxical nature of joy, emphasizing how joy is experienced both as a physical sensation and as an emotion, while also being a shared communal experience.

### Joy as a paradox

This subtheme explores the paradox of joy as described by participants: although joy was often experienced as fleeting and unpredictable, it frequently left a profound and enduring emotional impact. This contrast between transience and lasting effect formed the core paradox. While joy was also described as both physical and emotional, participants did not present this duality as contradictory, but rather as part of its richness and complexity. Participants frequently described joy as an elusive and fleeting feeling, often appearing unexpectedly. However, despite its transient nature, participants described joy as involving a reflective, cognitive process, where they made sense of the experience, revisited it in memory, or interpreted it as personally meaningful. This cognitive engagement often gave joy a lasting emotional impact. The sources of joy described were varied, including achieving goals, engaging in activities for the first time, appreciating arts and nature, and participating in community or spiritual practices. Joy was commonly found in perceived small, insignificant moments but was aligned with personal values and an aftermath of finding meaning and purpose.

… and it [joy] comes and it goes. And it’s just wonderful, isn’t it? It’s like butterflies and, but it is quite elusive, isn’t it? It does go up and down, and sometimes it comes for no, no reason. (P11)

I think it wasn’t kind of instantaneous. Like “I’m joyous, I’m going to dance”, it was kind of like about half an hour later, kind of like, oh, now I’m absolutely loving this. And then the day after and the weeks after … (P13)

### The physical and emotional symphony of joy

Participants described different types of joy and noted that joy was often accompanied by other positive emotions, such as pride, gratitude, excitement, and awe. Joy was also experienced physically, often described as a “filling up” or “overwhelm” in the chest, or as a rush of warmth and lightness throughout the body. Several participants spoke of tears, tingling sensations, or an urge to move, such as dancing, smiling uncontrollably, or physically embracing others. These embodied reactions often accompanied moments of intense connection, awe, or accomplishment, reinforcing the deeply somatic nature of joy. For some, the physical experience of joy was so powerful that it felt “like electricity” or “a bubbling up” that could not be contained. While sometimes active, joy could also be peaceful and tranquil, and participants frequently referred to it as more profound than happiness, describing it as “pure” or “utter” joy.

Happiness is just a general state […] when we talk about joy, we are talking about something elevated from that (P9)

… and erm, in nature and it’s like you on the top of a mountain and you look at, you look around and there’s all this absolute beauty and, and suddenly it sort of comes into your heart and it’s like utter joy. (P10)

In addition to physical and emotional sensations, several participants described joy as a mental experience—marked by a sense of inner clarity, calm, or perspective. For some, joy brought about a pause in mental noise or an alignment between thoughts and values. These mental states often followed moments of deep presence, suggesting that joy could foster not just feeling, but also reflective awareness and mental balance. Joy could also coexist alongside pain and sorrow, particularly during meaningful experiences such as grief, where fond memories brought both joy and sadness.

… and then Mum, Mum died. You couldn’t have asked for a better death, and I was with her, which is what I wanted. I wanted to be there. That was extremely painful and extremely joyful as well. (P4)

### The communal nature of joy

For many, joy was inextricably tied to communal sharing, where joy was enhanced through the joy of others. Empathetic joy, or vicarious joy, emerged as participants felt joy through the experiences of those around them. However, some expressed concerns that sharing joy on social media could diminish its meaning, especially if others failed to respond positively or if the joy felt vulnerable or misinterpreted in public spaces.

… but you see how joyful the others are and that can bring you joy. (P1)

… because joy’s also something you can pass on, I don’t think you can pass happiness on, but you can pass joy on. (P2)

These accounts illustrate how the act of sharing joy publicly, particularly in digital spaces, can introduce vulnerability or dilute the personal significance of the experience, especially when met with indifference or performative engagement.

## Determinants of joy: influences and barriers

This main theme aligns with the second objective of the study, which aims to explore the contextual and personal factors contributing to joy cultivation and its loss among the general adult population. The subthemes illustrate the various influences and barriers that impact the manifestation of joy. These include the impact of negativity and positivity, cultural and familial influences, the important role that connection plays, and individual differences in the way people navigate life’s challenges.

### The influence of positivity and negativity

Participants described how their capacity to experience joy was influenced by both positive and negative interactions and environments. Positive relationships, optimistic outlooks, and supportive social connections were seen as key facilitators of joy, while difficult relationships or pervasive negativity often diminished it. Conversely, joyful experiences increased within positive relationships and when spending time with optimistic individuals. A negative mindset, especially when dealing with painful emotions or challenges, hindered joy, while a positive, pragmatic attitude and broader perspective towards difficulties fostered moments of joy.

… if, you know, my child’s having a bad day or we have a bad exchange or my husband and I have a bad exchange, or something like that. It can take away from the possibility of joy. (P7)

And not letting it get to you, just the having a [… .] pragmatic sense of getting through that current obstacle to when a time when you’ll … . be able to be joyous or happy again. (P13)

### Cultural and familial influences

Family upbringing and cultural influences shaped participants’ expectations regarding what should bring joy and how or when it should be expressed. Caitlin recalls words said to her as a child, “ *… my mum used to say to me, ‘don’t expect to enjoy your life’.”* Some individuals suppressed their urge to express joy or negatively appraised their perceived inability to experience joy like others. Frequently, participants shared their belief that the media portrays a distorted depiction of joy, leading people to look for joy in significant life events, causing disappointment and disillusionment when these events also brought the stress and complications of real life. These media were seen to overlook the joy found in ordinary, daily occurrences, something participants deeply treasured.

I think we’re fooled into thinking what joy is. You know, we watch films, it’s the big things. But on the film, everything falls into place perfectly and the music you know, comes in at the right time, and you give the impression that that should be joy, when it’s not [… .] And if you’re trying to compare yourself to something that’s fake, you’re never gonna live up to it. So, you’re gonna be constantly disappointed and miss the real moments of joy. (P14)

### The role of connection

Participants consistently shared that connection was fundamental to their experiences of joy, emerging from connection with others and sharing values, meaning, and purpose. Being connected to oneself, especially the authentic self, and being open to a range of emotions, were deemed crucial for experiencing joy. Connection to nature and the environment was significant, particularly for those who felt disconnected from people. Feeling connected to something larger than oneself and engaging in loved activities such as hobbies and spiritual practices were important for experiencing joy. Periods of diminished joy coincided with a loss of connection in various ways.

What brings me Joy’s connection with other people and … doing things with and for other people that I love … That’s what brings me joy … (P8)

Joy for me is to do with being present, allowing the full spectrum of emotions. It’s also to do with knowing what guides you, your values. [… .] It [joylessness] feels like I’m, I’m not being me, not being authentic. I’m not really connecting to myself. (P4)

### Navigating life’s burdens

Participants observed that diminished joy often occurred during times of stress or worry, stemming from diverse responsibilities and high expectations. These responsibilities and expectations often led to a perceived lack of freedom. Some attributed this burden to adulthood, noting that joy seemed easier for children. Experiencing poverty in a society of abundance fostered feelings of entrapment and lack of autonomy. Insufficient sleep, excessive workload, and fatigue, as well as mental health challenges like depression, were all related to reduced joy. Instances of joy were associated with relinquishing responsibilities or inhibitions and tapping into one’s “inner child,” alongside the capacity to nurture gratitude for moments of joy after facing adversity.

You just, you just prioritise that [responsibilities] over joy and not just because, just because life overtakes you … (P1)

I process it [joy] as in, I will sometimes just recognise that I just feel … oh [sighs] really just really … ugh … nothing’s bothering me. Nothing. Yeah, just, just quiet and calm. Perhaps nothing pressing to do. No expectations of anything on me at that moment in time. (P6)

## Impact of joylessness: coping behaviours

This main theme explores the psychological impact of diminished joy and the coping responses participants engaged in as a result, as well as how joyful experiences contributed to long-term emotional balance and resilience. Participants reflected that recognizing and experiencing moments of joy contributed to their enduring happiness, wellbeing, and resilience. The two sub-themes illustrate what life feels like without joy, the coping behaviours employed in response, and how memories of joy have a lasting impact on mental health.

### Coping behaviours in response to joylessness

Participants described life as bleak and monotonous during periods of diminished joy, contrasting this state with the vitality and wellbeing brought by joy. The absence of joy often triggered coping mechanisms, ranging from positive, constructive behaviours—such as seeking out joyful company, connecting with nature, or engaging in hobbies—to negative responses, including excessive alcohol consumption and withdrawal.

… things feel really bleak when you don’t have moments of joy. It makes everything else seem harder. (P8)

… to be able to recognise moments of joy and be aware that they exist in your life improves your sense of wellbeing and your sense of worth. (P14)

Participants also noted that sometimes their coping behaviours were unconscious and reactive. For example, Dean described how during a joyless period, they found themselves increasingly isolated and drinking more, without realizing how these behaviours were a direct response to their emotional state.

Erm, so I was, I was sort of by myself a lot and not [… .] getting involved in things. [… .] Yeah, probably just er, drank a lot more, but yeah. (P5)

### Joy’s enduring influence: mental health and memory

The lasting influence of joyful experiences on mental health was frequently discussed by participants, who described how moments of joy provided them with emotional balance and stability. Participants likened joy to a sustaining force that could fuel resilience and foster long-term mental wellbeing. The positive impact of joy extended beyond the moment of experience, as participants recalled and relived joyful memories during challenging times.

I was not hypervigilant, I wasn’t anxious [… .] I felt so much more balanced inside after having a weekend of true joy. (P7)

Joy was described as energizing and empowering, leaving participants feeling more open, confident, and hopeful. For many, memories of joy acted as a buffer against future hardship, with photographs or other tangible reminders serving as triggers for reliving joyful moments. This connection between memory and joy was highlighted as a powerful tool for sustaining mental health.

… it kind of opens up your mind [… .] and it’s a moment you keep in your mind for the rest of your life [… .] reliving it brings the same emotions, so it wasn’t just for that moment. (P3)

## Strategies for cultivating joy

This theme explores practical methods for enhancing joy among adults in the UK, addressing the fourth objective of the study: to investigate how joy can be cultivated within the community. Participants identified several strategies that can foster joy, emphasizing the importance of joy education, meaningful conversations about joy, and revitalizing community connections.

### Joy education

Participants advocated for the inclusion of joy cultivation within mental health programmes, suggesting that teaching individuals how to experience and express joy is as important as learning to manage negative emotions. They reflected on how cultural and familial influences during upbringing affected their ability to experience joy, and how joy education could help children and adults alike develop a greater capacity for joy. In workplaces, participants stressed the need for voluntary, self-directed engagement in joy-promoting activities, as forced participation often led to feelings of artificial joy and disappointment.

… in every workplace, it’s about doing more, doing better, but none of that’s joyful [… .] We need programmes that genuinely help people find joy in their everyday lives. (P9)

… everyone needs the freedom to step in and out of whatever brings them joy, rather than being pushed into situations where others might find joy but they don’t. (P5)

### Conversations about joy

Participants found that engaging in conversations about joy, particularly during the interviews, was deeply therapeutic, helping them to reflect on and relive joyful moments. These conversations heightened their awareness of joy and reminded them of its importance in their lives. Participants suggested that such dialogues should be encouraged more widely, including in public forums and media outlets, to foster a broader appreciation of joy.

This conversation has reminded me that I need joy [… .] you get so bogged down in responsibility that you forget about it. This interview sparked a bit of joy for me. (P11)

Participants were critical of how the media frequently diminishes joy, noting that news outlets often prioritize negative stories, and when positive events are reported, they tend to feel tokenistic. Participants expressed a desire to see media portrayals of joy that reflect everyday, meaningful moments, such as the quiet joy of reconnecting with family after a long absence, acts of community kindness, or stories of resilience in the face of hardship. For example, one participant suggested that local news segments showcasing neighbours supporting each other or small, real-life accomplishments could feel more genuine and emotionally resonant than overly polished or scripted depictions of success. Participants called for more authentic representations of joy in the media, which could promote a cultural shift towards recognizing and celebrating joy in everyday life.

### Revitalising community connections

Participants believed that government and local councils should invest in community groups and activities that foster connections and bring people together over shared interests. The loss of community spaces, such as youth centres and Sure Start programmes (UK-based early childhood and family support initiatives), was lamented as a barrier to joy. Participants emphasized that these spaces were vital for promoting joy by fostering a sense of belonging and providing opportunities for meaningful interactions. Financial constraints were seen as another obstacle, with participants advocating for government support to make joy-promoting activities more accessible.

… having something to look forward to with meaning behind it, giving young people a chance, like youth centres [… .] is essential. We need spaces that allow people to connect and find joy. (P13)

I think that shared understanding helps people get back to joy [… .] It’s about getting back to basics, spending time with family, and finding joy in those simple, meaningful moments. (P12)

## Discussion

This study explored how adults in the UK experience, perceive, process, cultivate, and lose joy in everyday life. The insights gained provide a valuable understanding of the mechanisms underlying joy, highlighting opportunities to nurture joy across diverse settings. Fourteen adults shared detailed accounts of their joy experiences, elucidating the circumstances that foster or hinder joy. The findings highlight joy’s unique emotional, cognitive, and social dimensions, while suggesting practical strategies to cultivate joy for broader well-being and resilience. This discussion reviews key findings and addresses the implications for practice, research, and policy.

Participants described joy as a distinct and intense emotion, often more immediate and vivid than happiness. While some considered joy more profound in its emotional impact, others reflected on its fleeting nature in contrast to the broader, more sustained quality of happiness. This aligns with previous research suggesting that joy is associated with meaning, values, and a sense of fulfilment (Damasio, 2003; Watkins et al., 2018). The physical sensations of joy reported by participants, such as a “filling up” or feeling of warmth, support the notion of joy as a somatic experience tied to physiological homoeostasis (Damasio, 2003). Joy’s entanglement with other positive emotions, including gratitude and love, reinforces the “crowded conceptual space” of joy (Davis et al., 2020), emphasizing the need to further delineate its unique characteristics and associations with other emotions.

The experience of joy during painful moments, particularly grief, aligns with research indicating that joy can coexist with sorrow (Ishizu & Zeki, 2017), although this remains underexplored in empirical studies. Understanding how joy manifests during suffering could offer new insights into emotional resilience and post-traumatic growth, an area ripe for further investigation (King, 2020; Underwood, 2020). Participants also noted the fleeting nature of joy, often beyond their control, yet lasting through memory. Memory played a significant role in sustaining joy, suggesting the potential for interventions that harness joyful recollections to bolster well-being during difficult times (Fredrickson, 2001). This finding highlights the need for future research into the role of memory in the enduring impact of joy, which could inform positive psychology interventions (PPIs) aimed at enhancing long-term mental health.

Participants consistently identified positive relationships as central to joy cultivation. This mirrors existing literature on the role of social connectedness in well-being (Johnson, 2020b; Watkins, 2020), suggesting that joy thrives in environments where empathy, trust, and shared experiences are fostered. The concept of *Mudita*, or vicarious joy, rooted in Buddhist traditions, was echoed in participants’ experiences of sharing in others’ joy, providing a cross-cultural perspective on joy that is less recognized in Western psychological frameworks (Casioppo, 2020). Cultivating *Mudita* through appreciative joy meditation has been shown to enhance subjective well-being (Gu et al., 2022), and could be integrated into well-being programmes.

Connection to nature emerged as a critical source of joy, offering participants space for introspection and restoration. This finding aligns with evidence linking nature exposure to reductions in negative affect and increases in positive emotions (McMahan & Estes, 2015). Connection to one’s authentic self was also foundational for joy, supporting theories of eudaimonic well-being, where living in alignment with core values fosters flourishing (Deci & Ryan, 2008; Watkins et al., 2018). This reinforces Van Cappellen’s (2020) notion that joy is deeply connected to personal meaning and identity. Participants’ descriptions of joy through creativity and art further align with research on the role of creative expression in human flourishing (Cloninger & Cloninger, 2020; Underwood, 2020).

Negative relationships and environments were found to significantly hinder joy, corroborating findings that social comparison, envy, and rivalry dampen joy (Gilbert et al., 2014). Participants’ reluctance to share joy on social media due to distrust or scepticism reflects the growing body of research on the negative effects of social media on well-being (Zell & Moeller, 2018). Negative media portrayals, pressure to meet societal expectations, and financial constraints further contributed to joylessness, underscoring the importance of addressing systemic and cultural factors that limit joy. Adulthood was perceived as a burden, with responsibilities and anxieties leaving little space for joy. This reflects Vaillant’s (2008) assertion that adults often struggle to express strong emotions such as joy, which may stem from childhood experiences of emotional suppression (Gilbert et al., 2014). Interventions aimed at fostering emotional literacy and positive emotional expression, particularly in adult populations, could help mitigate these barriers and promote joy.

The study found that periods of diminished joy were characterized by lethargy and negativity, reinforcing the role of joy in sustaining overall happiness and mental health (Lyubomirsky et al., 2005). Participants reported actively engaging in strategies to cultivate joy during these times, such as connecting with nature, seeking positive relationships, and reminiscing about past joyful moments. These proactive coping strategies align with Fredrickson’s (2001) broaden-and-build theory, which posits that positive emotions like joy expand cognitive resources and foster resilience. Participants’ unconscious substitution of joy with pleasure-seeking behaviours, such as overeating or excessive alcohol consumption, highlights the need for interventions that help individuals differentiate between short-term pleasure and enduring joy (Cabanac, 2017).

Participants advocated for the inclusion of joy cultivation in mental health programmes, noting that existing interventions often focus solely on alleviating negative emotions. PPIs such as the PERMA model (Seligman, 2011), which emphasizes positive emotion, engagement, relationships, meaning, and accomplishment, could serve as a foundation for promoting joy. Research has shown that PPIs improve subjective well-being, making them a valuable tool for mental health initiatives (Appiah et al., 2020; Bolier et al., 2013).

The workplace was identified as a key area for intervention, with participants emphasizing the importance of autonomy in fostering authentic joy. Leaders can play a crucial role in creating joyful work environments, as evidenced by research showing that joyful workplace cultures reduce burnout and enhance productivity (Gabbay & Barrett, 2020). Strengths-based micro-coaching—brief, targeted coaching sessions focused on identifying and enhancing an individual’s personal strengths—may also be effective in promoting personal attributes conducive to joy (Peláez et al., 2020). For example, participants described joyful workplaces as those that allowed flexibility, recognized employee strengths, and fostered informal moments of connection, such as shared laughter, spontaneous celebrations, or the freedom to take breaks in nature or quiet spaces. These small but meaningful conditions were seen as supporting emotional balance and a sense of being valued.

Participants frequently described experiencing joy through nature, creative expression, and periods of respite from responsibilities. These findings align with research indicating the mental health benefits of natural environments and creative activities (Cloninger & Cloninger, 2020; McMahan & Estes, 2015). However, it is important to acknowledge that access to these forms of joy may be disproportionately available to individuals with greater economic resources and social capital, potentially framing joy as not only an emotional experience but also a by-product of privilege. Structural barriers such as financial hardship, lack of leisure time, or limited access to natural environments and creative outlets may significantly constrain opportunities for joy among socioeconomically disadvantaged populations. Future research and policy must therefore consider how societal inequalities shape access to joy, ensuring interventions aiming at promoting joy do not inadvertently reinforce existing disparities.

### Practice, research, and policy implications

The findings from this study hold significant implications for practice, research, and policy. Practically, the study offers insight into how joy cultivation could be meaningfully integrated into mental health programmes, well-being initiatives, and community-based interventions. For example, participants emphasized the value of reflective conversations about joy, suggesting that therapy and group-based interventions could incorporate prompts or narrative exercises focused on recalling and recognizing moments of joy. Participants also advocated for greater autonomy in joyful experiences, suggesting that well-being programmes should offer flexible, strengths-based activities that individuals can tailor to their interests. Practices such as nature-based walks, gratitude journaling, or small creative expressions could be embedded as low-cost, scalable ways to foster joy. Mental health professionals might also consider using visualization or memory-based interventions that help clients reconnect with past joyful experiences, reinforcing emotional resilience.

This aligns with the growing body of PPIs that aim to enhance wellbeing through intentional practices (Rashid & Seligman, 2018; Seligman, 2011). In clinical and community settings, practitioners can develop tools and interventions that teach individuals to recognize, express, and cultivate joy, particularly in everyday life contexts where stress and adversity are common (Fredrickson, 2013). From a research perspective, the study underscores the need for further qualitative investigations that explore how joy is experienced across diverse cultural, socioeconomic, and clinical contexts, and how it interacts with other emotional states such as gratitude, awe, and contentment. Future studies could also examine how individuals define and sustain joy over time, particularly in the face of adversity or life transitions. Future research could also explore the potential for developing reliable experimental methodologies for inducing and measuring joy, a noted challenge in the field. Finally, at the policy level, the study suggests the necessity of advocating for joy education within schools, workplaces, and communities. Policy initiatives should focus on promoting social connections, community programmes, and educational curricula that foster emotional resilience, social bonding, and overall wellbeing (Davidson & Schuyler, 2015). This could be instrumental in enhancing collective mental health and mitigating the widespread effects of stress and emotional burnout.

### Limitations and future research

This study has limitations related to sampling strategy and participant demographics. The use of convenience sampling via social media platforms may have introduced self-selection bias, attracting individuals who were already inclined to reflect on emotional well-being or who had more flexible time to participate, potentially skewing the sample towards a particular demographic profile. Notably, the sample was overwhelmingly female (12 out of 14 participants) and predominantly White British (13 out of 14), which limits the transferability of findings to more diverse populations. The gender imbalance may have shaped the thematic emphases, particularly on communal connection and emotional expression, while the ethnic homogeneity may have overlooked culturally distinct understandings or expressions of joy. We also acknowledge that the phrasing and framing of the recruitment material on social media may have resonated more with women or individuals from majority cultural backgrounds. Future studies should adopt purposive or stratified sampling strategies to ensure greater gender and ethnic diversity, thereby enriching understanding of joy across sociocultural contexts.

Additionally, while qualitative research prioritizes the exploration of subjective meaning-making, it is important to acknowledge that individual differences, such as personality traits, emotional regulation styles, coping mechanisms, and life circumstances, may have significantly shaped participants’ experiences and narratives of joy. These personal variables, although not the primary focus of this study, may have functioned as confounding factors, influencing how joy was recalled, interpreted, and expressed. For instance, individuals high in trait optimism or openness to experience may be more inclined to report or reflect on joyful experiences, while those experiencing ongoing adversity or emotional fatigue may struggle to access or articulate joy. Future research could integrate psychometric assessments or mixed-method designs to explore how such individual-level characteristics interact with lived experiences of joy.

Although income data were not directly collected, participants’ socio-economic status was inferred from occupation data, which suggested a predominantly moderate- to high-income sample. As such, while poverty and financial strain were thematically referenced by some participants, the study did not include sufficient representation across income levels to permit meaningful subgroup analysis by economic status. Future studies should consider collecting direct income or socio-economic indicators to better explore how financial realities shape experiences of joy. Lastly, while the primary researcher conducted the initial coding and theme development, the data analysis process was strengthened through peer review. Specifically, the second author cross-checked the analytical procedures and reviewed the coherence between emergent themes, subthemes, and supporting quotes to ensure consistency and interpretive accuracy. However, the interview recordings were not independently listened to or coded by a second researcher. This introduces a potential risk of interpretive bias, and future studies may benefit from multiple coders or independent audits to further enhance analytical rigour.

## Conclusion

The findings from this study provide valuable insights into how adults in the UK experience, cultivate, and lose joy in their everyday lives, highlighting joy as a complex emotion deeply intertwined with overall happiness, wellbeing, and resilience. Joy was understood to manifest not only in major life events but also in seemingly insignificant moments, often linked to connection with others, nature, and one’s authentic self. However, the study also revealed barriers to joy, such as societal pressures, negative relationships, and emotional burnout. These findings offer insight into how joy cultivation could be supported within mental health and wellbeing programmes, and how environments, whether in communities, workplaces, or schools, might be shaped to foster emotional resilience and social connection. Future research should explore specific strategies for inducing and sustaining joy across different demographic groups and contexts, including examining joy in challenging life circumstances. It is recommended that policymakers and practitioners promote joy education and community-based initiatives that enhance social bonds and provide individuals with the tools to intentionally cultivate joy, which could have lasting impacts on individual and collective wellbeing.

## Supplementary Material

Table S1.docx

## Data Availability

De-identified data presented in this manuscript will be shared upon reasonable request and receipt of a completed data request form.
