# Supplementary material for: The complexities of joy: a qualitative study of joy cultivation, loss of joy, and happiness in British adults
Source: Int J Qual Stud Health Well-being. 2025 May 24;20(1):2508946. doi: 10.1080/17482631.2025.2508946 (PMC12107667; doi:10.1080/17482631.2025.2508946)
Supplement: Table S1.docx [file ZQHW_A_2508946_SM5550.docx]

**Table S1.** Semi-structured interview guide

| **Question #/aim** | **Interview Question**  *Follow-up probes* |
| --- | --- |
|  |  |
| **1** | **Can you describe a specific moment that you experienced joy and happiness in your life? How did you process and express that joy?** |
|  | *Follow-up probes*: In your opinion, how might cultural or societal influences shape an individual's understanding and expression of joy? |
| **2** | **Reflecting on your daily life, what personal habits or activities do you believe contribute most to your experience of joy?** |
|  | *Follow-up probes*: How do social connections and relationships impact your ability to cultivate and sustain joy? |
| **3** | **Think about a time when you felt a loss or diminishment of joy. What were the circumstances surrounding that experience?** |
|  | *Follow-up probes*: How do societal expectations or pressures contribute to the loss of joy for individuals? |
| **4** | **Can you discuss how moments of joy, or the loss of joy impact your overall sense of happiness in life?** |
|  | *Follow-up probes:* How do you navigate through periods of diminished joy without compromising your overall happiness? |
| **5** | **From your perspective, what societal or cultural changes could enhance the overall joy and well-being of adults in the United Kingdom?** |
|  | *Follow-up probes:* Are there specific policies or initiatives you believe would be effective in promoting joy at a societal level? Can you share an example of a community or group that has successfully fostered joy? What elements or practices contributed to that success? |
